# A novel laparoscopic renal denervation system in a preclinical swine model

**DOI:** 10.1038/s41598-026-43593-7

**Published:** 2026-03-07

**Authors:** Linwei Zhao, Wei Yang, Binbin Zhu, Yahui Liu, Peng Tian, Xinyu Guo, Yang Dong, Zhiqiang Fan, Huiping Li, Yu Feng, Lijie Zhu, Minfu Bai, Qiuping Zhao, Xingkun Zhang, Chenchen Si, Qianqian Cheng, Ganxin Yan, Chuanyu Gao

**Affiliations:** 1 Department of Cardiology, Zhengzhou University People’s Hospital, Henan, China; 2 Department of Cardiology, Fuwai Central China Cardiovascular Hospital, Henan, China; 3 Henan Key Laboratory of Coronary Heart Disease Control & Prevention, Henan, China; 4 Department of General Surgery, Fuwai Central China Cardiovascular Hospital, Henan, China; 5 Department of Pathology, Fuwai Central China Cardiovascular Hospital, Henan, China; 6 Department of Urology, Henan Provincial People’s Hospital, Henan, China; 7 Department of Hypertension, Fuwai Central China Cardiovascular Hospital, Henan, China; 8 Department of Laboratory Medicine, Fuwai Central China Cardiovascular Hospital, Henan, China; 9 Lankenau Institute for Medical Research and Lankenau Medical Center, Wynnewood, PA USA; 10 Sidney Kimmel Medical College, Thomas Jefferson University, Philadelphia, PA USA

**Keywords:** Laparoscopic Renal Denervation, Radiofrequency Ablation, Renal Nerve Ablation, Hypertension, Diseases, Medical research, Nephrology

## Abstract

Laparoscopic renal denervation (RDN) represents a specialized approach for hypertension, particularly when conventional methods face limitations. While catheter-based RDN has demonstrated significant efficacy in recent large-scale clinical trials and has been integrated into international guidelines, its effectiveness can be constrained by complex renal anatomy or incomplete ablation. We developed a novel laparoscopic RDN system, comprising an integrated radiofrequency (RF) clamp, RF generator, and cooling pump, to achieve more controlled adventitial ablation. This study aims to evaluate the safety, feasibility, and optimal parameters of this new system in a preclinical swine model. Sixteen pigs were divided into an immediate group (*n* = 10) for power optimization and a 28-day follow-up group (*n* = 6). An optimal setting of 10 W for 10 s was identified, balancing effective nerve injury with minimal vascular damage. In the 28-day follow-up group, this setting was associated with a significant reduction in systolic blood pressure (median 125.5 to 109.0 mmHg, *p* = 0.010) and serum norepinephrine (217.56 to 170.47 ng/L, *p* = 0.017). Our novel laparoscopic RDN system demonstrates feasibility and safety, providing a potential rescue strategy or supplementary therapy for specific patient populations, such as non-responders to endovascular treatment or those with complex vascular anatomy.

**Supplementary Information:**

The online version contains supplementary material available at 10.1038/s41598-026-43593-7.

## Introduction

Hypertension remains a paramount global health challenge and a leading risk factor for cardiovascular diseases, affecting an estimated 1.13 billion people worldwide. Despite the wide range of antihypertensive medications available, around 10–15% of patients develop resistant hypertension. Resistant hypertension is defined as blood pressure that remains high despite using three or more antihypertensive medications, including a diuretic, placing patients at risk for serious cardiovascular and cerebrovascular events.^1–3^.

The sympathetic nervous system plays a key role in hypertension by increasing renal sodium retention, boosting renin release, and causing vasoconstriction. Renal denervation (RDN), which targets sympathetic overactivity by disrupting renal nerves, has emerged as a promising treatment for resistant hypertension.

While early trials yielded variable results, recent high-quality, sham-controlled trials have provided robust evidence for the efficacy of multi-electrode radiofrequency^4,5^ and ultrasound devices^6–8^. Consequently, RDN has been integrated into several international guidelines as a complementary treatment for resistant hypertension.^9,10^.

**Fig. 1:**
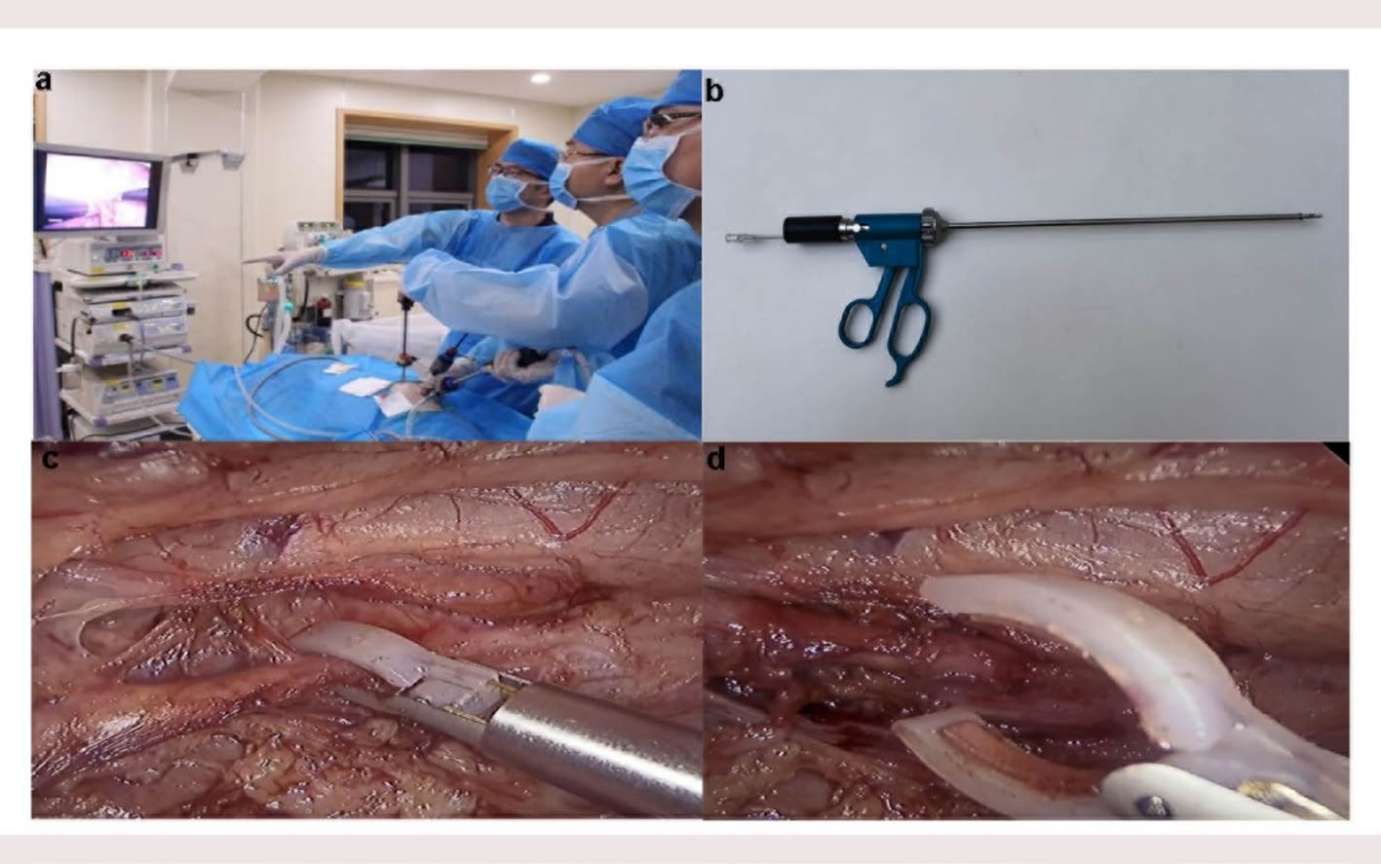
Laparoscopic RDN Procedure. (**a**) A view of the RDN team during an operation, utilizing laparoscopic techniques with a monitor displaying the procedure. (**b**) The RFA clamp used in the procedure. (**c**) Intraoperative image showing the insertion of a laparoscopic tool for tissue manipulation. (**d**) The clamp head and electrode pads applied to the renal artery.

Despite these advancements, individual variability in response persists, which may be attributed to the inherent challenges of achieving consistent, uniform circumferential ablation in patients with complex renal artery anatomy or varying vessel wall thickness. To overcome these limitations, we have developed a laparoscopic RDN approach (Fig. 1). This system has been specifically engineered to achieve more comprehensive and uniform circumferential ablation while utilizing potentially smaller, yet highly effective, energy release compared to some conventional approaches. The design aims to optimize energy delivery from the adventitial surface, thereby overcoming the challenges of incomplete ablation and variability associated with previous RDN modalities, **a**nd provides a potential rescue or alternative strategy for specific patient populations, such as non-responders to endovascular treatment or those with complex vascular anatomy. The primary aim of this preclinical study in a porcine model is therefore to rigorously evaluate the safety profile and feasibility of this novel laparoscopic RDN system. While acknowledging the more invasive nature of the laparoscopic approach compared to catheter-based interventions, we specifically assessed whether direct adventitial visualization could minimize vascular injury while ensuring effective nerve ablation. Specifically, we seek to determine its potential to achieve effective renal sympathetic nerve ablation while preserving renal function.

## Results

### Immediate group(*n* = 10)

The Immediate Group focused on evaluating the short-term effects of laparoscopic renal nerve denervation (RDN) using different RF power settings (8 W, 10 W, 12 W, 14 W, 16 W), each applied for 10 s to the proximal, mid, and distal segments of the renal artery. This group aimed to assess the immediate vascular and nerve damage, providing valuable insights into the procedural effects of RDN at varying power levels.

### Vascular Changes: (Fig. 2&Table S1)

**Fig. 2:**
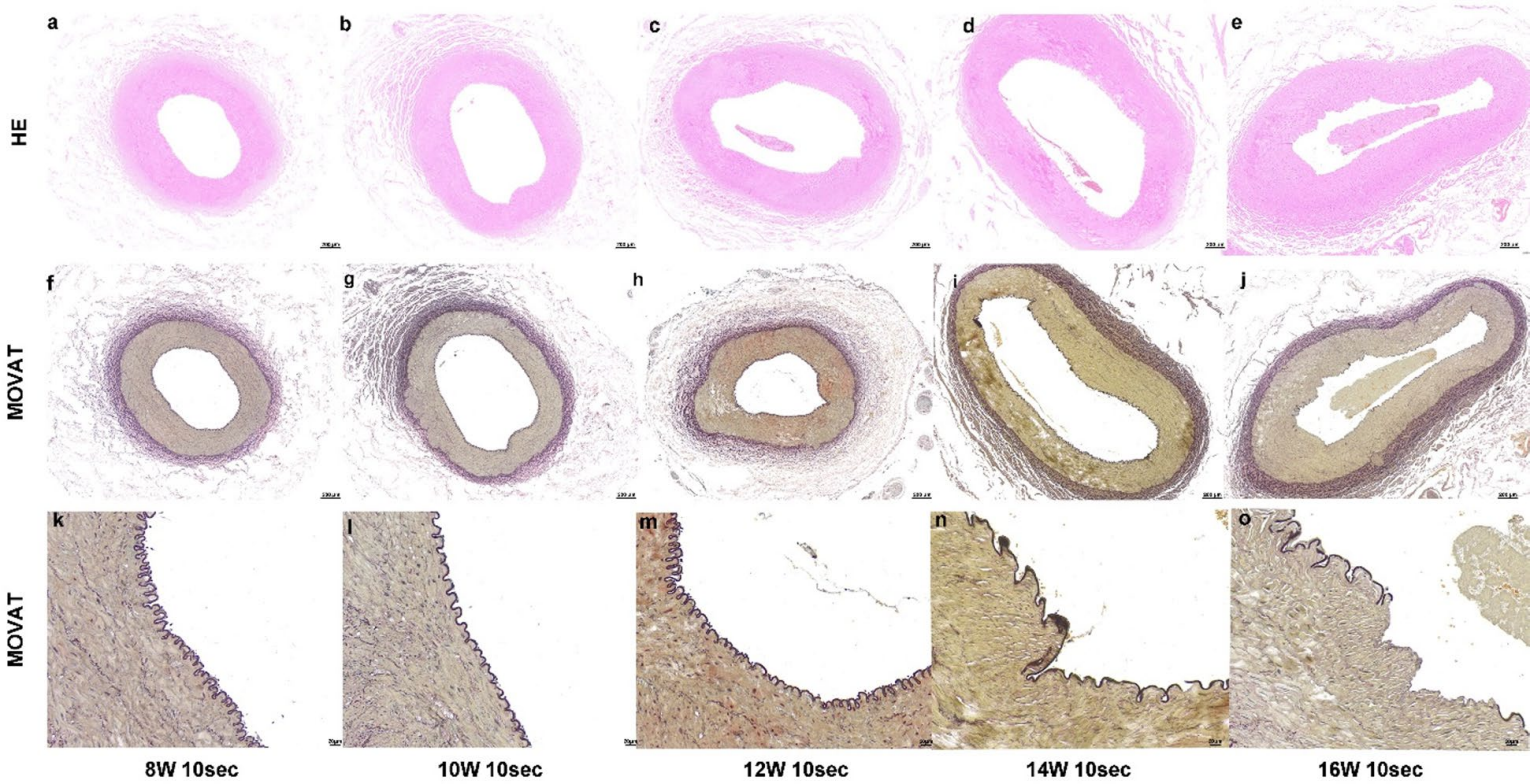
Histopathological images of renal artery sections at different RF power settings (8 W, 10 W, 12 W, 14 W, 16 W) applied for 10 s. HE staining (a-e) and MOVAT staining (f-j) were performed to assess the effects of different power settings on renal artery structure. Images (a-e) show the changes in the arterial wall and smooth muscle with increasing RF power. Panels (k-o) display higher magnification MOVAT staining, highlighting the degree of tissue damage and vascular changes at different power levels.

At 8 W RF (a, f, k): The internal elastic lamina remained intact without visible tears. Smooth muscle cells in the tunica media were tightly arranged, with only minor vacuolization observed. The adventitia showed no significant changes.

At 10 W RF (b, g, l): The internal elastic lamina remained structurally intact, with no visible tears. Minor vacuolization was noted in the tunica media, but the overall structure remained well-preserved. Mild loosening of the tunica adventitia was observed, though no significant damage was evident.

At 12 W RF (c, h, m): The internal elastic lamina showed broadening of its undulations without major tears. Smooth muscle cell disorganization and areas of necrosis were visible in the tunica media. Mild loosening of the adventitial fibers was observed, suggesting early-stage tissue damage.

At 14 W RF (d, i, n): Significant damage was observed in both the internal elastic lamina and tunica media, with pronounced tearing and structural breakdown. Extensive loosening and tissue damage were evident in the tunica adventitia.

At 16 W RF (e, j, o): Severe damage to the vascular layers, particularly the internal elastic lamina and tunica media, with substantial tearing and destruction. The tunica adventitia showed widespread loosening and extensive tissue damage.

### Nerve Damage (Fig. 2&Table S1)

**Fig. 3:**
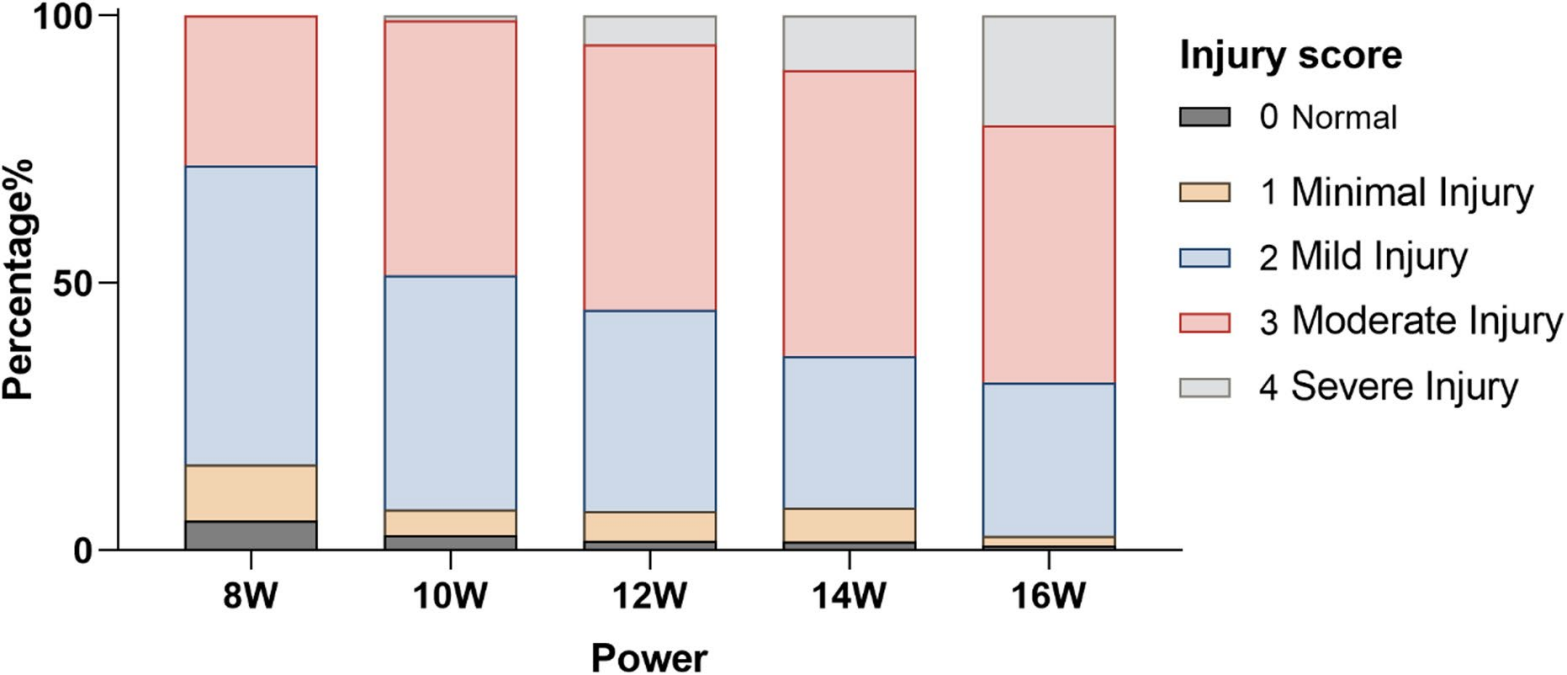
Frequency distribution of injury scores at different energy levels. The stacked bar chart illustrates the percentage of ablation sites (*n* = 12 sites per power setting, derived from 2 pigs, 2 renal arteries per pig, and 3 ablation sites per artery) exhibiting different grades of nerve injury immediately following RDN at energy levels of 8 W, 10 W, 12 W, 14 W, and 16 W for 10 s.

The extent of nerve damage immediately after RFA varied depending on the RF power, as shown by the injury scores (Fig. 3). At 8 W, injury scores were relatively low, with most of the nerve tissue exhibiting minimal to mild injury, characterized by mild vacuolization and largely intact nerve structure. However, as the RF power increased to 10 W and 12 W, there was a noticeable increase in moderate injury (red) and mild injury (blue), particularly at the mid and distal ablation sites. At these power levels, although severe injury (gray) was still relatively low, the percentage of moderate injury steadily increased.

As the RF power was further increased to 14 W and 16 W, injury scores rose significantly, especially at mid and distal sites, with a marked increase in severe injury (gray). At 16 W, the nerve damage was extensive, with high levels of vacuolization, necrosis, and severe structural breakdown. The distribution of injury severity shows a clear pattern of increasing nerve damage with higher RF power, particularly at higher RF power settings where the proportion of severe injury reached its peak.

### Follow-up group(*n* = 6)

The Follow-Up group evaluated the effects of the laparoscopic RDN procedure over a 28-day period. This group aimed to assess the persistence of vascular integrity, nerve changes, blood pressure trends, and renal function post-procedure. Based on the results from the immediate group, we selected the 10 W for 10 s setting to examine its 28-day impact.

### Renal artery angiography(Fig. 4)

**Fig. 4:**
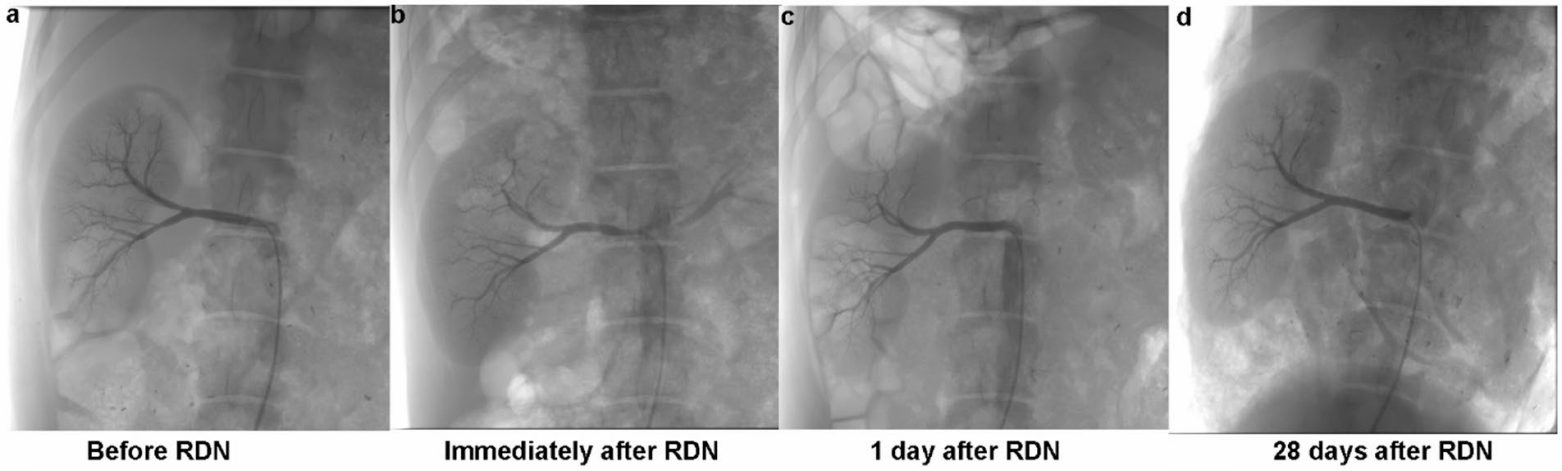
Renal artery angiography at different time points (10 W, 10s). (**a**) Renal angiography before RDN. (**b**) Immediately after RDN, showing the status of the renal artery. (**c**) One day after RDN, illustrating the post-procedure vascular condition. (**d**) Twenty-eight days after RDN, demonstrating the long-term vascular integrity.

Immediately after the RDN procedure, renal artery spasms were observed in all animals. Renal artery angiography performed 1 day post-ablation confirmed that the renal arteries were patent, with no significant stenosis, thrombosis or dissection. The vessels showed normal blood flow, indicating that the procedure did not result in any lasting structural impairment. At the 28-day follow-up, angiography conducted in the 6 experimental animals also showed no significant narrowing or other abnormalities in the renal arteries, further supporting the long-term safety of the laparoscopic RDN procedure in preserving vascular integrity.

### Pathological results

**Fig. 5:**
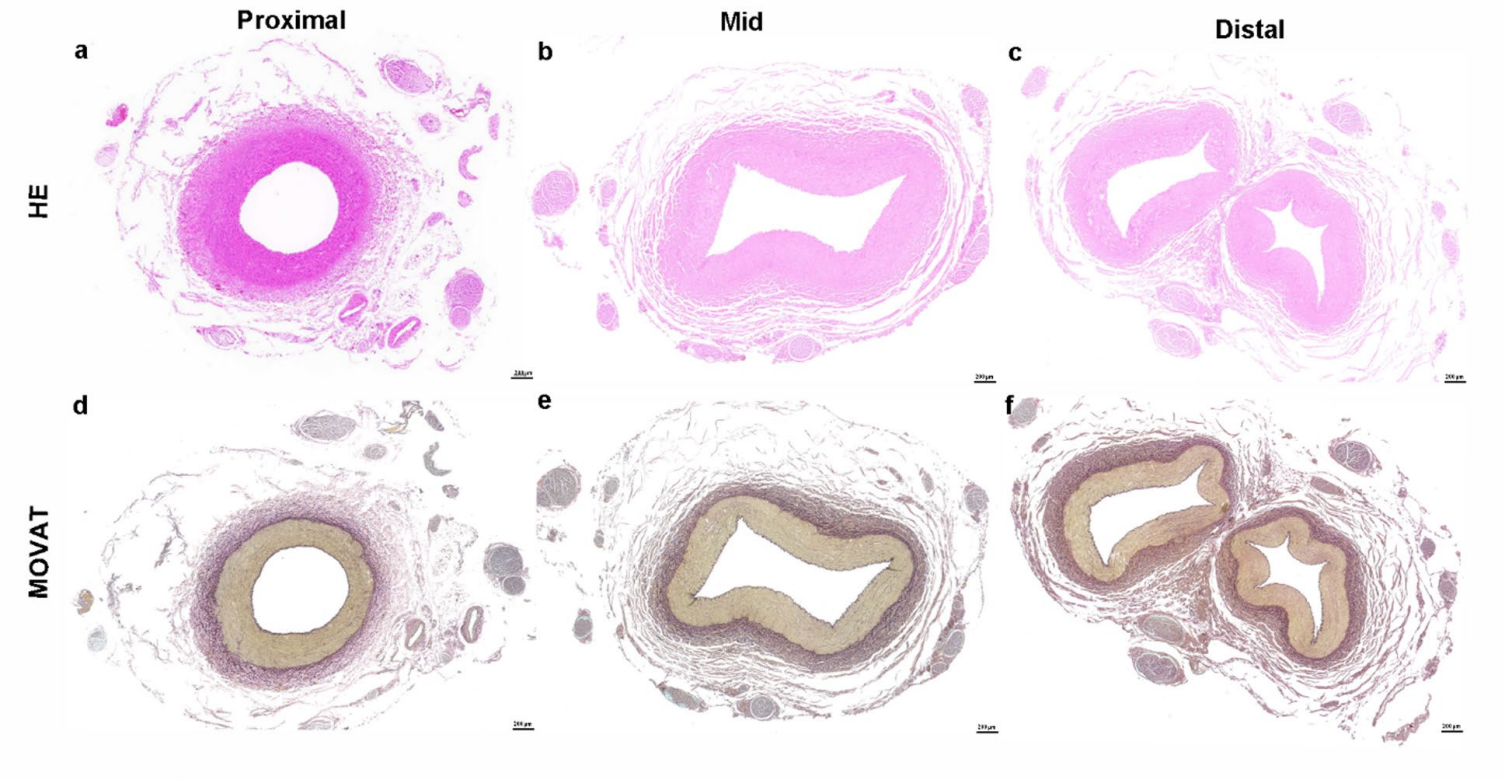
Histopathological images of renal artery sections (10w, 10s). HE (**a-c**) and MOVAT (**d-f**) staining of the proximal (a, d), mid (b, e), and distal (c, f) segments of the renal artery after RDN treatment.

The histopathological analysis of RFA demonstrated significant effects on 360-degree perivascular nerve structures across different anatomical locations (proximal, mid, and distal segments). While the vascular structure remained intact across all regions, the degree of nerve disorganization, vacuolization, and degeneration increased progressively from the proximal to the distal segments. (Fig. 5)

### Vascular wall changes over 28 days: (Fig. 6)

**Fig. 6:**
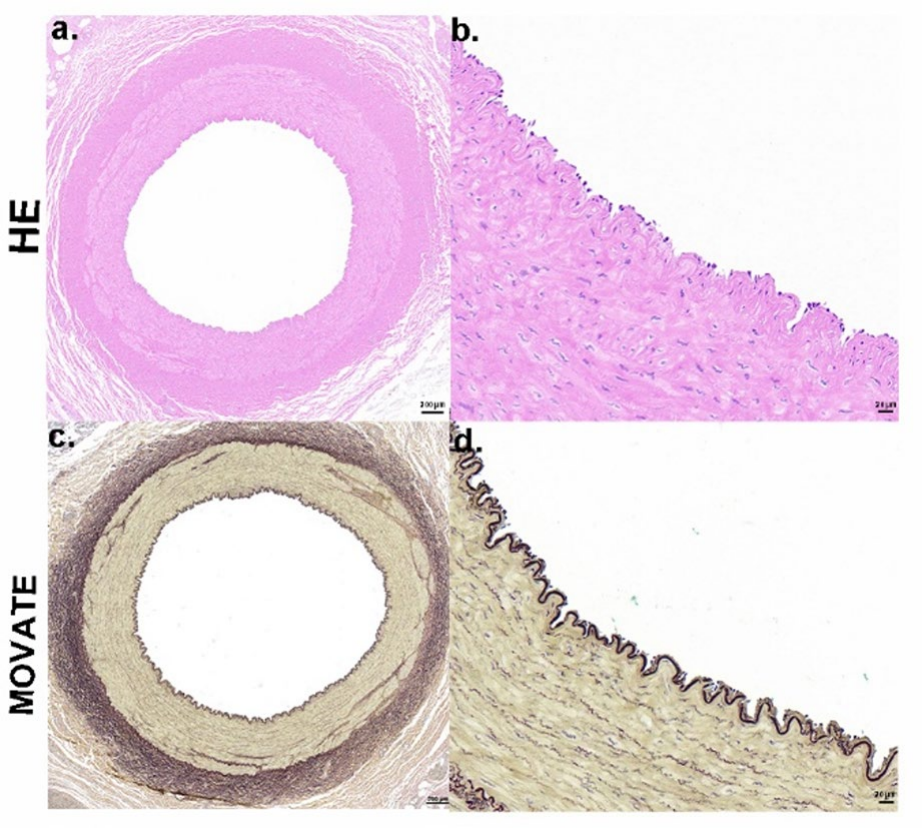
Representative histopathological images of renal artery Sect.  28 days following RDN. Representative images of HE (**a**,** b**) and MOVAT (**c**,** d**) staining of renal artery walls 28 days post-RDN.

At 10 W RF power applied for 10 s, followed by a 28-day post-ablation period, histopathological evaluation showed that the internal elastic lamina and the arterial wall remained intact, with no visible tears or damage. Minor vacuolization in the smooth muscle layer suggested a tissue response, but the overall structure remained well-preserved. The adventitia showed mild loosening, but no significant damage to the vasculature was observed. This suggests that, 28 days after the procedure, the integrity of the arterial wall and internal elastic lamina was effectively maintained, with no severe structural damage to the artery.

### Nerve changes over 28 days (Fig. 7)

**Fig. 7:**
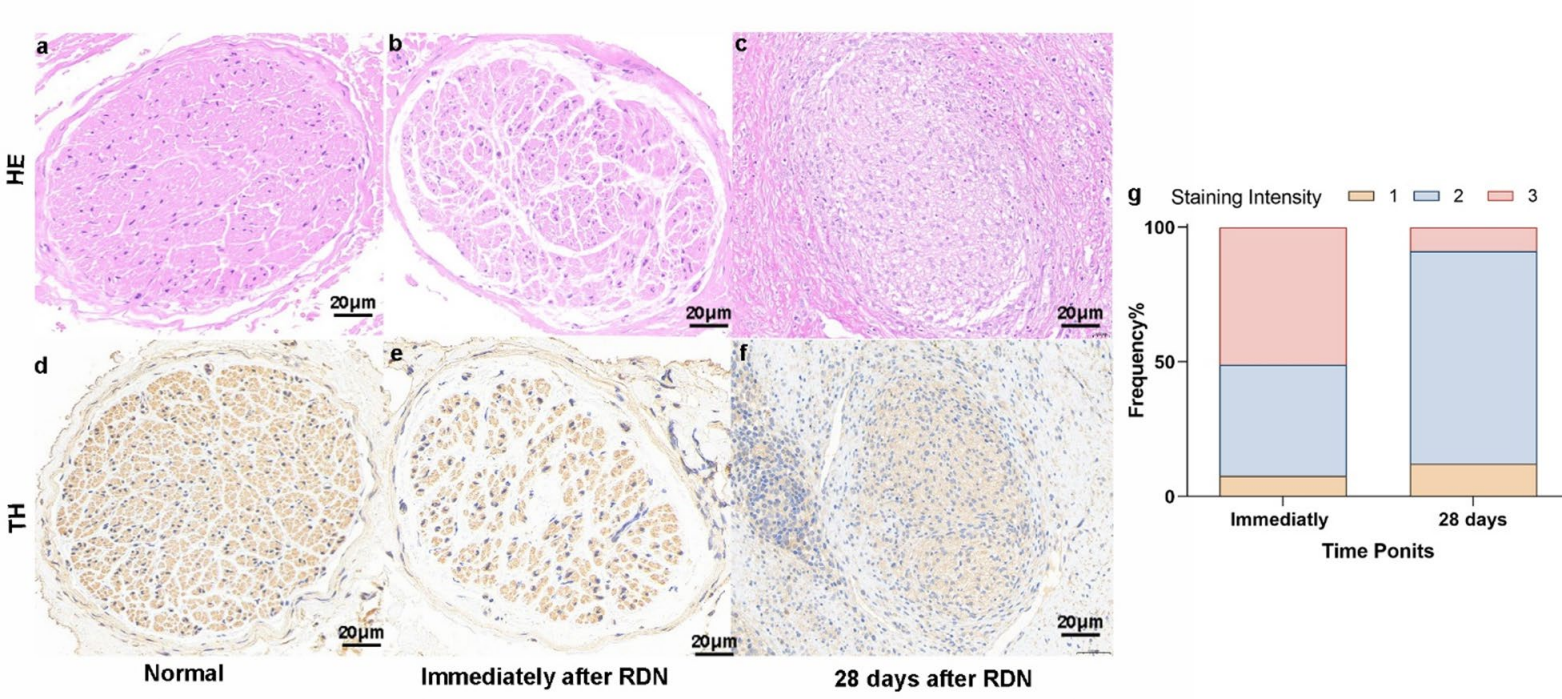
Representative histopathological images of renal nerve damage 28 days after RDN. HE (**a-c**) and TH (**d-f**) staining of renal nerve bundles. Panels show progressive nerve damage as indicated by histopathological changes and decreased TH expression following RDN. (**g**) Stacked bar chart shows the IHC staining score of renal nerve.

Immediately after the ablation, HE staining showed changes in the nerve structure, suggesting initial damage. However, no noticeable change in TH expression was observed at this early stage. By 28 days post-ablation, HE staining indicated significant disruption of the nerve microstructure, with further breakdown and damage. TH staining showed a notable decrease in expression, reflecting a reduction in nerve fiber integrity and function. These findings highlight the extent of long-term damage to the nerve, with both structural and functional deterioration becoming more evident over time.

### Blood pressure trends over 28 Days(Fig. 8a & Table S2)

**Fig. 8:**
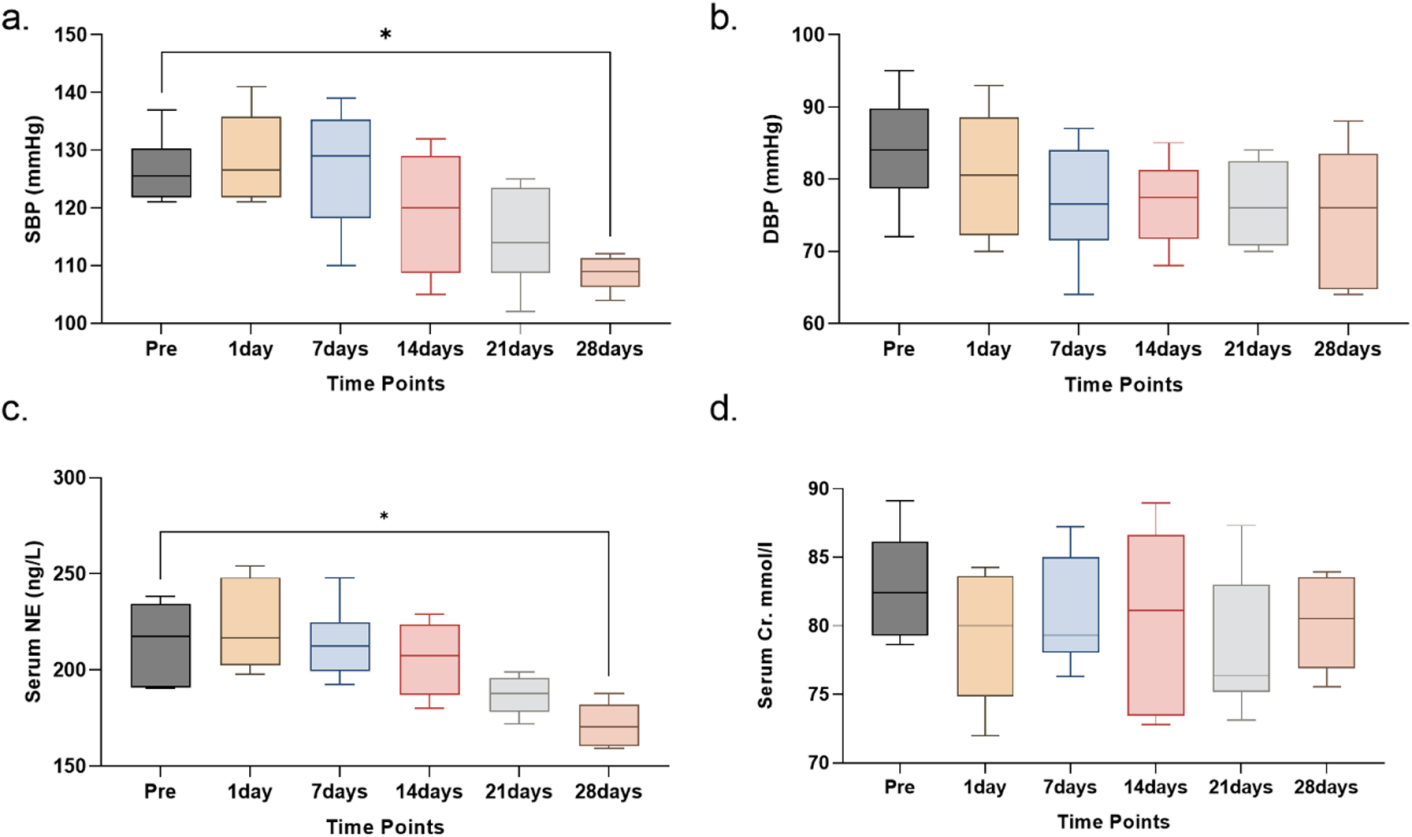
Blood pressure, serum NE, and serum creatinine changes following RDN.(*n* = 6). (**a**) SBP measured at the indicated time points. A significant reduction in SBP was observed at 21 days and 28 days compared to baseline (Pre). (*p* < 0.05). (**b**) DBP exhibited a downward trend that did not reach statistical significance (*p* = 0.224 vs. baseline). (**c**) NE levels at different time points. Serum NE levels showed a gradual decline from baseline (Pre) to 28 days, with a significant reduction observed at 28 days (*p* < 0.05). (**d**) Serum Cr levels measured at various time points. Serum Cr levels remained stable after an initial decrease at day 1, with a slight rise observed at 28 days, but no significant differences were found between time points.

SBP and DBP were measured at multiple time points.

SBP: Starting from a baseline median of 125.5 mmHg (IQR 122.0-128.0), SBP showed a slight initial increase during the first week, then progressively decreased. By day 28, SBP was significantly reduced to a median of 109.0 mmHg (IQR 107.0-111.0; *p* = 0.010 vs. baseline).

DBP: Baseline median DBP was 84.0 mmHg (IQR 81.0–88.0). DBP decreased initially and remained below baseline levels throughout the follow-up period, with a median of 76.0 mmHg (IQR 65.0–82.0) at day 28; this reduction was not statistically significant (*p* = 0.224 vs. baseline).

### NE trends over 28 Days (Fig. 8c & Table S2)

Serum NE levels, with a baseline median of 217.56 ng/L (IQR 190.89-232.98), showed a progressive decrease over the 28-day period. At day 28, NE levels were significantly reduced to a median of 170.47 ng/L (IQR 160.71-180.23; *p* = 0.017 vs. baseline).

### Peripheral blood creatinine changes (Fig. 8d & Table S2)

Creatinine levels were monitored to assess renal function. Starting from a baseline median of 82.40 µmol/L (IQR 79.49–85.16), Cr levels remained stable throughout the 28-day follow-up (e.g., day 28 median 80.54 µmol/L, IQR 77.33–83.41; *p* > 0.9999 vs. baseline). No significant changes were observed at any time point, indicating preserved renal function.

## Discussion

This preclinical study establishes the safety and biological feasibility of a novel laparoscopic RDN system in a porcine model, as an exploratory proof-of-concept. The sympathetic nervous system is a key contributor to the pathogenesis of hypertension^11^. Using an optimized 10 W/10s setting, we achieved successful sympathetic modulation, evidenced by a triad of findings: (1) significant reduction in systemic NE (Table S2), (2) histopathological evidence of perivascular nerve ablation (reduced TH expression) (Fig. 7), and (3) a significant reduction in SBP at 28 days. Crucially, these effects were achieved without compromising renal artery integrity or renal function, demonstrating a favorable safety profile within the context of minimally invasive surgery.

Our parameter optimization study revealed a critical dose-response relationship and safety trade-off that contextualizes these findings (Table S1, Figs. 2 and 3). While 12 W induced quantitatively superior nerve damage, it significantly increased the risk of medial necrosis. Consequently, we selected 10 W as a conservative parameter prioritizing vascular safety. This safety-oriented approach resulted in moderate rather than severe nerve necrosis. This deliberate moderation likely explains why the trend in DBP reduction did not reach statistical significance (*p* = 0.224), highlighting the fine balance between maximizing efficacy and ensuring safety in surgical denervation.

Our findings align with and build upon previous research in the field. Our team’s prior explorations with laparoscopic RDN, including studies in obesity-induced hypertension models and clinical reports^12^, laid the foundation for the current, more advanced system. Similarly, Baik et al.^13^ reported a laparoscopic RDN system that achieved neural density reduction and acute blood pressure lowering. While their reported acute SBP reduction of 9.55 mmHg is noteworthy, our study demonstrates a significant chronic reduction in SBP at 28 days, alongside a substantial decrease in NE, providing evidence for a more sustained effect with our system. However, direct comparisons remain challenging due to differences in animal models, ablation protocols, and follow-up durations.

Placing these results in the broader context, the landscape of RDN has evolved with recent high-quality trials, such as the SPYRAL^4,5^ and RADIANCE^6–8^ series, leading to its inclusion in the ESH and ESC guidelines^9,10^. However, despite the proven efficacy of endoluminal systems, achieving consistent circumferential ablation remains challenging in certain anatomies due to the deep adventitial location of nerves or the “heat-sink” effect of renal blood flow. In endovascular approaches, high-velocity renal blood flow dissipates thermal energy from the vessel wall, preventing the formation of transmural lesions sufficient to reach deep perivascular nerves^14,15^. Current endovascular radiofrequency systems typically operate within a strictly limited lower power range of 6 to 8 Watts to minimize endothelial thermal injury^16^, which can be insufficient to penetrate deeper adventitial layers in the presence of high-velocity blood flow. Our laparoscopic system was designed to address these specific physical limitations. By utilizing a visually-guided approach to target nerves directly from the adventitial space, our system avoids the cooling interference of luminal blood flow and facilitates potentially more uniform ablation.

While acknowledging the greater invasiveness of laparoscopy compared to catheter-based methods, this system fills a critical clinical niche as a complementary strategy for specific patient populations. It is particularly applicable for: (1) patients with complex renal anatomy (e.g., multiple accessory arteries, severe tapering, or early branching) that precludes optimal catheter-to-wall contact; (2) patients with severe chronic kidney disease where avoiding contrast media is a clinical priority; and (3) non-responders to endovascular treatment where anatomical constraints or the heat-sink effect may have limited ablation efficacy. To further mitigate surgical trauma, the system is compatible with single-port laparoscopy and robotic-assisted platforms, offering superior precision and potentially shorter recovery times^17,18^. Furthermore, while the suggestion that laparoscopic RDN could be combined with other abdominal procedures is technically intriguing, we acknowledge that this clinical application remains currently speculative and would require rigorous multidisciplinary coordination.

### Limitations

We acknowledge several limitations in this feasibility study. First, the observed reduction in SBP (*p* = 0.010) in our normotensive model should be interpreted as a physiological consequence of sympathetic modulation rather than direct anti-hypertensive evidence. The findings are derived from a modest sample size (*n* = 6) (Table S2), and thus require validation in larger, hypertensive cohorts. Moreover, our 28-day follow-up, while providing valuable mid-term insights, is insufficient to address the long-term durability of denervation or the potential for functional nerve re-growth, which remains a key concern for clinical translation. However, it is hypothesized that the surgical perivascular dissection inherent to our laparoscopic approach may physically disrupt the neural regenerative scaffold, theoretically reducing the likelihood of nerve regeneration compared to endovascular techniques, although this warrants confirmation in longer-term studies.

In conclusion, this study establishes our laparoscopic RDN system as a safe and biologically feasible platform. It represents a valuable potential supplement to existing RDN strategies, offering a refined avenue for treating complex resistant hypertension. Future research in chronic hypertensive models is imperative to confirm its long-term therapeutic potential.

## Methods

### Novel laparoscopic rdn system: design features and composition

This study used a newly designed laparoscopic RDN system, carefully engineered to deliver precise and controlled radiofrequency (RF) energy. The overarching design goal is to effectively ablate renal sympathetic nerves while critically minimizing the risk of vascular damage, a significant step forward in RDN technology. The system’s strength lies in the seamless integration of three key, synergistically functioning components:

(1) Radiofrequency Ablation (RFA) Clamp: The RFA clamp is distinguished by its intuitive control handle, which allows for precise opening and closing maneuvers, ensuring the renal artery is securely yet gently encircled. This meticulous design is paramount for the efficient transmission of both RF energy and cooling saline directly to the target area. The clamp head houses high-quality platinum alloy bilateral electrodes (9 mm x 2 mm), chosen for their excellent conductivity and durability, which translates to stable and reliable energy delivery during the procedure. A crucial design feature is the bilateral electrode configuration. This ensures even and circumferential energy distribution around the renal artery, a key factor in achieving effective, predictable, and controlled renal sympathetic nerve ablation while reducing the likelihood of hot spots or untreated areas. The effective clamp rod length of 370 mm also offers ample reach for various anatomical presentations.

(2) RF Generator: The system incorporates a specialized RF generator that acts as the intelligent core of the energy delivery process. It expertly controls energy delivery by dynamically modulating RF power based on real-time feedback from tissue impedance and electrode temperature sensors (with a target temperature of 55–60 °C). The sophisticated temperature-control algorithm optimizes energy transfer, ensuring consistent lesion formation while preventing common issues like overheating, tissue charring, and steam pops. This intelligent control directly contributes to both the safety and efficacy of the procedure.

(3) Cold Saline Infusion Pump: Complementing the RF generator, the cold saline infusion pump provides an adjustable saline flow rate (10–40 ml/min). This active cooling mechanism is vital to prevent the electrodes from overheating during energy application. By meticulously maintaining the electrode temperature within the optimal range of 55–60 °C, the system effectively protects the surrounding delicate vascular tissues from unintended thermal damage and ensures that the RF energy is precisely targeted at the renal sympathetic nerves, enhancing the specificity of the ablation.

This integrated system, where the circumferential RFA clamp (with its advanced electrodes and temperature sensing) works in concert with active cooling from the saline infusion pump and the intelligent, real-time adjustments of the RF generator, is specifically designed to achieve uniform, predictable, and highly controlled circumferential ablation. This holistic design approach is intended to significantly enhance both the safety and efficacy of RDN when compared to traditional methods, addressing many of their known limitations. (Fig. 1)

### Animals and experimental design

All experimental procedures were approved by the Ethics Committee of Animal Management and Use Committee of Huizhi Yinghua Medical Technology Research and Development (Shanghai) Co. Ltd. and were conducted in accordance with the American Physiological Society’s “Guides for the Care and Use of Laboratory Animals” published by the National Institutes of Health. This study, reported in compliance with the ARRIVE guidelines, involved 16 adult male Yorkshire pigs (average weight: 82.73 ± 5.82 kg), which were purchased from Shanghai Jiagan Biotechnology Co., Ltd. (Shanghai, China). All pigs were housed in a controlled environment with regulated temperature (20–24 °C) and humidity (40–60%) on a 12:12-h light-dark cycle, with ad libitum access to standard chow and water. The 16 pigs were randomly assigned to either the immediate group (*n* = 10) or the 28-day follow-up group (*n* = 6). This assignment was performed using a computer-generated random allocation sequence by a staff member not involved in the experimental procedures or assessments. In the immediate group, RDN was performed targeting three distinct sites (proximal, mid, and distal segments) along each accessible main renal artery. To ensure standardized treatment parameters, animals presenting with accessory renal arteries were excluded from this study. At each site, RF energy was applied once using one of five power settings (8 W, 10 W, 12 W, 14 W, or 16 W), each for a fixed duration of 10 s. Two pigs were allocated to each power setting, with both renal arteries in each animal treated. The optimal RF parameter for the follow-up study was selected based on histopathological findings from this group, aiming to achieve effective nerve damage, while inducing minimal acute vascular injury.

Renal angiography was performed before and immediately after the RDN procedure to assess acute changes in the renal artery lumen. Following the final angiography, animals in this group were humanely euthanized via intravenous injection of euthasol, and their renal arteries were harvested for histopathological examination.

The follow-up group consisted of 6 pigs and was designed to assess the effects of RDN after 28 days. In this group, RDN was performed using the optimal power and duration parameters determined from the immediate group (10 W for 10 s). Norepinephrine (NE) levels were measured using high-performance liquid chromatography with electrochemical detection (HPLC-EC) in plasma samples, which provides a sensitive and specific method. Serum creatinine (Cr) levels were monitored using an automated biochemistry analyzer to assess renal function over time. Blood samples were collected at multiple time points: pre-surgery, 1 day, 7 days, 14 days, 21 days, and 28 days post-surgery. After 28 days, the animals were euthanized, and their renal arteries were collected for further histopathological examination. This experimental design allowed for the evaluation of both the immediate effects of RDN and its impact after 28 days on the renal arteries and biochemical markers.

### Anesthesia and surgical preparation

For anesthesia, the pigs were initially sedated with intramuscular injections of ketamine hydrochloride (5 mg/kg), midazolam (0.5 mg/kg), and atropine (0.5 mg). Following the establishment of intravenous (IV) access, anesthesia was induced using propofol (3 mg/kg), sufentanil (1 µg/kg), and vecuronium bromide (0.1 mg/kg). Sevoflurane (0.5-1.5MAC) was then used to maintain anesthesia throughout the procedure. All surgical procedures were performed under sterile conditions.

### Renal artery angiography

Baseline renal artery angiography was performed prior to the RDN procedure to assess the initial anatomical status and blood flow of the renal arteries. In the immediate group, a second angiographic evaluation was conducted immediately after the RDN procedure to assess any changes in the renal artery structure and blood flow dynamics. For the follow-up group, renal artery angiography was conducted at multiple time points: pre-surgery, immediately post-surgery, 1 day post-surgery, and just before euthanasia at the end of the 28-day follow-up period. The purpose of these angiographic assessments was to evaluate both the immediate and longer-term effects of RDN on the renal artery morphology and function.

### RDN procedure

The pigs were positioned in a lateral decubitus position to optimize access to the renal arteries. Three small abdominal incisions were made for trocar and laparoscopic instrument insertion. Pneumoperitoneum was established using CO₂ insufflation, creating the necessary pressure to maintain an inflated abdominal cavity, which facilitated optimal visualization and working space for the laparoscopic procedure. Once the peritoneal cavity was insufflated, laparoscopic instruments were introduced to expose the renal arteries. Laparoscopic forceps were used to gently lift and stabilize surrounding tissues, improving access to the renal arteries. After adequate exposure, the laparoscopic RDN procedure was performed, targeting the renal sympathetic nerves with precision. The procedure was closely monitored to prevent vascular injury, with careful manipulation of the renal arteries to avoid damaging surrounding structures. The procedure was completed under continuous anesthesia, with no complications observed. The protocol was adapted from previous studies^19^ to ensure consistency with well-established methodologies.

For the RDN procedure, a laparoscopic RFA clamp, connected to an RF generator and a cold saline infusion pump, was introduced into the retroperitoneal space through a 10-mm trocar. The clamp was positioned to deliver RF energy to the proximal, mid, and distal segments of the renal artery. RF energy was applied in discrete bursts at 8 W, 10 W, 12 W, 14 W, and 16 W for 10 s each.

During the RFA process, temperature was meticulously controlled to ensure the electrode temperature did not exceed 60 °C, while tissue impedance was maintained below 300Ω.

### Post-ablation tissue collection and processing

Immediately following the ablation procedure, animals in the Immediate group were humanely euthanized via intravenous injection of euthasol. The renal arteries were then harvested, rinsed with physiological saline to remove blood and impurities, and fixed in 10% formalin solution. After fixation, the tissues were embedded in paraffin, and sections were prepared for staining using Hematoxylin and Eosin (H&E) and MOVAT pentachrome staining methods. These stains were used to assess the impact of the ablation on the renal arteries and surrounding tissues.

### Nerve damage evaluation

Nerve damage in the renal artery nerve bundles (proximal, mid, and distal regions) was assessed using a semi-quantitative grading system based on histopathological changes^20^. H&E and MOVAT pentachrome staining allowed the examination of the nerve structure at different injury levels. The grading system for nerve damage was as follows: (Fig. S1)

Grade 0: Normal: No injury is observed, and the nerve fibers remain intact.

Grade 1: Minimal Injury: Slight vacuolization with minimal inflammation, nerve fibers remain largely intact.

Grade 2: Mild Injury: Prominent vacuolization, occasional pyknotic nuclei, nerve structure relatively intact.

Grade 3: Moderate Injury: Significant vacuolization, frequent pyknotic nuclei, partial disruption of nerve structure.

Grade 4: Severe Injury: Extensive vacuolization, necrosis, and severe loss of nerve architecture.

### TH Immunohistochemistry staining for renal nerves

Immunohistochemical staining for tyrosine hydroxylase (TH) was carried out to assess the changes in sympathetic nerve activity, particularly in the nerve fibers. TH serves as a marker for sympathetic nerve integrity, as it plays a key role in catecholamine synthesis. The staining intensity was semi-quantitatively analyzed using ImageJ software, which helped assess the extent of TH expression in the nerve fibers. The intensity was categorized as follows:

Grade 0: No staining or faint staining, indicating no detectable nerve fibers.

Grade 1: Low intensity staining, indicating minimal nerve fiber presence.

Grade 2: Moderate intensity staining, indicating a moderate amount of nerve fibers.

Grade 3: High intensity staining, indicating abundant nerve fibers.

For the follow-up group, angiography was performed 4 weeks post-procedure to assess vascular integrity and tissue changes.

### Renal artery damage evaluation

Renal artery damage following laparoscopic renal denervation (RDN) was assessed by evaluating endothelial cell loss, thrombus formation, and medial damage. Thermal injury leads to smooth muscle cell (SMC) loss/necrosis and replacement with proteoglycan or fibrous tissue. The damage was examined using H&E and MOVAT pentachrome staining methods, providing insights into the vascular injury and healing process. This evaluation identifies the extent of vascular damage, highlighting the procedure’s impact on renal artery integrity.

The grading and analysis were carried out by two experienced pathologists, who were blinded to the treatment allocation and independently conducted separate assessments to ensure consistency.

### Statistical analysis

Descriptive statistics were used to summarize the data. Appropriate inferential statistical tests were applied based on the data distribution, with non-parametric tests being used for data with a non-normal distribution. Specifically, the Friedman test was used to assess overall differences across time points, and Dunn’s multiple comparisons test was used for pairwise comparisons following a significant Friedman test result. All P values were two-tailed, with *P* < 0.05 considered statistically significant. All statistical analyses were conducted using GraphPad Prism software (version 9.5; GraphPad Software Inc., San Diego, CA, USA).

## Electronic supplementary material

Below is the link to the electronic supplementary material.

Supplementary Material 1

## Data Availability

The data will be made available upon request to the corresponding authors.

## Abbreviations

RDN: Renal Denervation
RF: Radiofrequency
SBP: Systolic Blood Pressure
DBP: Diastolic Blood Pressure
NE: Norepinephrine
Cr: Creatinine
RFA: Radiofrequency Ablation
TH: Tyrosine Hydroxylase
SMC: Smooth Muscle Cells
