# Supplementary material for: A novel laparoscopic renal denervation system in a preclinical swine model
Source: Sci Rep. 2026 Mar 7;16:10533. doi: 10.1038/s41598-026-43593-7 (PMC13036002; doi:10.1038/s41598-026-43593-7)
Supplement: Supplementary file 1 — Supplementary Material 1 [file 41598_2026_43593_MOESM1_ESM.docx]

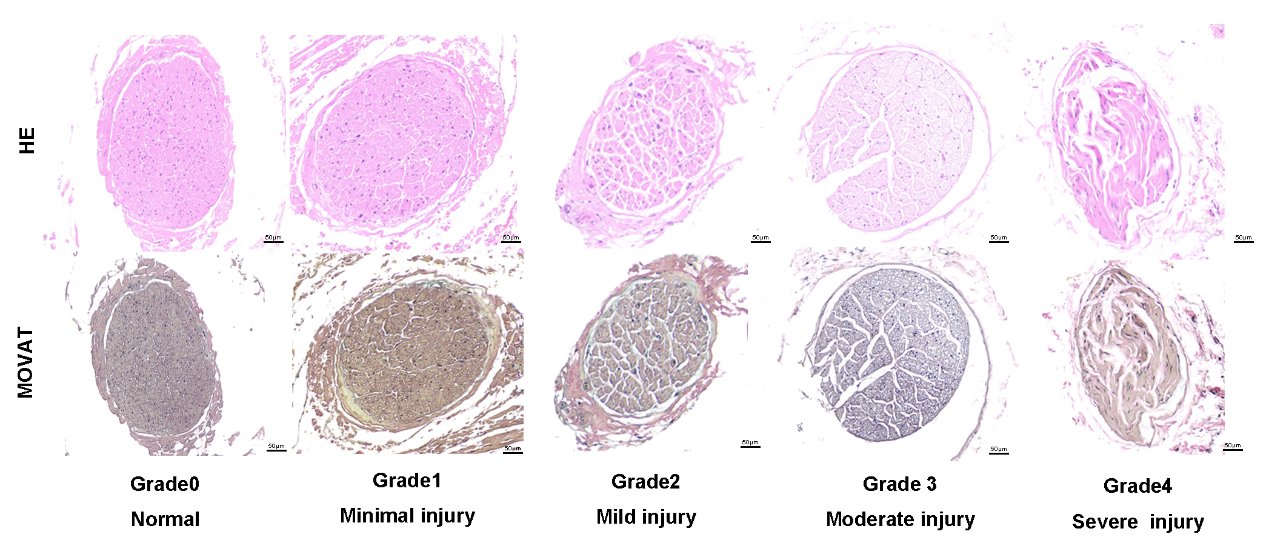
Fig S1. Histopathological grading of renal artery nerve damage in the immediate group. Representative images for each grade: (a, f) Grade 0: Normal structure, No injury is observed, and the nerve fibers remain intact. (b, g) Grade 1: Minimal injury. Slight vacuolization with minimal inflammation is observed, while the nerve fibers remain largely intact. (c, h) Grade 2: Mild injury. Prominent vacuolization, occasional pyknotic nuclei, nerve structure relatively intact. (d, i) Grade 3: Moderate injury. Significant vacuolization and frequent pyknotic nuclei are observed, with partial disruption of the nerve structure. (e, j) Grade 4: Severe injury. Extensive vacuolization, necrosis, and severe loss of nerve architecture are

Table S1. Summary of Nerve Injury and Vascular Integrity by RF Power Setting

| Power Setting (W) | Sample Size (N)  (Pigs/Arteries/Sites/Nerves fibers) | Nerve Injury Grade, % | | | | | Integrity of Internal Elastic Lamina | Smooth Muscle Cells of  Tunica Media | Changes in Tunica Adventitia |
| --- | --- | --- | --- | --- | --- | --- | --- | --- | --- |
|  |  | 0 | 1 | 2 | 3 | 4 |  |  |  |
| 8W | 2/4/12/125 | 5.60 | 10.40 | 56.00 | 28.00 | 0.00 | Remained intact | Minor vacuolization | No significant changes |
| 10w | 2/4/12/105 | 2.86 | 4.76 | 43.81 | 47.62 | 0.95 | Remained intact | Minor vacuolization | Mild loosening |
| 12W | 2/4/12/129 | 1.83 | 5.50 | 37.61 | 49.54 | 5.50 | Broadening of undulations, no tears | Disorganization, focal necrosis | Mild loosening |
| 14W | 2/4/12/127 | 1.57 | 6.30 | 28.35 | 53.54 | 10.24 | Significant damage, tearing | Significant damage, with tearing and structural breakdown | Extensive loosening and tissue damage |
| 16W | 2/4/12/112 | 0.89 | 1.79 | 28.57 | 48.21 | 20.54 | Severe damage, destruction | Severe damage, with tearing and destruction | Widespread loosening and extensive tissue damage |

Table S2. Blood Pressure, Serum Norepinephrine, and Serum Creatinine Levels at Baseline and Serial Follow-up Time Points After Laparoscopic Renal Denervation (n = 6)

|  | **SBP (mmHg)** | | **DBP (mmHg)** | | **NE (ng/L)** | | **Cr (µmol/L)** | |
| --- | --- | --- | --- | --- | --- | --- | --- | --- |
|  | Median (Q1, Q3) | p-value^‡^ | Median (Q1, Q3) | p-value^‡^ | Median (Q1, Q3) | p-value^‡^ | Median (Q1, Q3) | p-value^‡^ |
| Friedman Test |  | 0.001 |  | 0.256 |  | 0.001 |  | 0.577 |
| Pre | 125.5 (122.0, 128.0) | - | 84.0 (81.0, 88.0) | - | 217.56 (190.89, 232.98) | - | 82.40 (79.49, 85.16) | - |
| 1 day | 126.5 (122.0, 134.0) | > 0.999 | 80.5 (73.0, 87.0) | > 0.999 | 216.72 (203.83, 246.04) | > 0.999 | 80.02 (75.82, 83.41) | 0.8246 |
| 7 days | 129.0 (121.0, 134.0) | > 0.999 | 76.5 (74.0, 83.0) | > 0.999 | 207.98 (201.43, 217.22) | > 0.999 | 79.32 (78.64, 84.25) | > 0.999 |
| 14 days | 120.0 (110.0, 128.0) | > 0.999 | 77.5 (73.0, 80.0) | > 0.999 | 207.35 (189.35, 221.83) | > 0.999 | 81.13 (73.67, 85.85) | > 0.999 |
| 21 days | 114.0 (111.0, 123.0) | 0.154 | 76.0 (71.0, 82.0) | 0.320 | 187.85 (180.26, 194.71) | 0.224 | 76.39 (75.84, 81.55) | 0.320 |
| 28 days | 109.0 (107.0, 111.0) | 0.010* | 76.0 (65.0, 82.0) | 0.224 | 170.47 (160.71, 180.23) | 0.017 | 80.54 (77.33, 83.41) | > 0.999 |

n = 6; Dunn’s multiple comparisons test. ^‡^ vs. Baseline
